# Cement Industry Pollution and Its Impact on the Environment and Population Health: A Review

**DOI:** 10.3390/toxics13070587

**Published:** 2025-07-14

**Authors:** Alina Bărbulescu, Kamal Hosen

**Affiliations:** 1 Department of Civil Engineering, Transilvania University of Brașov, 5 Turnului Str., 500152 Brașov, Romania; 2 School of Ocean and Civil Engineering, Shanghai Jiao Tong University, Shanghai 200240, China

**Keywords:** cement manufacturing, greenhouse emissions, water pollution, environmental impact, population health

## Abstract

The cement industry, a foundation of global infrastructure development, significantly contributes to environmental pollution. Key sources of pollution include dust emissions; greenhouse gases, particularly carbon dioxide; and the release of toxic substances such as heavy metals and particulate matter. These pollutants contribute to air, water, and soil degradation and are linked to severe health conditions in nearby populations, including respiratory disorders, cardiovascular diseases, and increased mortality rates. Noise pollution is also a significant issue, inducing auditory diseases that affect most workers in cement plants, and disturbing the population living in the neighborhoods and fauna behavior. This review explores the pollution paths and the multifaceted impacts of cement production on the environment. It also highlights the social challenges faced by communities, underscoring the urgent need for stricter environmental policies and the adoption of greener technologies to mitigate the adverse effects of cement production on both the environment and human health.

## 1. Introduction

Concrete is the second most utilized material (about 30 billion tons yearly) in terms of consumption after water. Its widespread use is largely due to its high compressive strength, durability, and superior mechanical properties compared to other construction materials [1,2,3,4]. It is also preferred in architecture for its versatility (allowing for the creation of unique, modern, and minimalist designs with clean lines and innovative forms) [5,6] and the possibility of adapting to surroundings. These qualities transform it into an aesthetically appealing material in contemporary and sustainable design projects [7].

Concrete manufacturing relies upon more than 4 billion tons of cement produced annually. Concrete is composed of cement, fine and coarse aggregates, and water. Cement is the primary binding agent of concrete and provides it the strength that enables structures to withstand loads [8,9]. Ordinary Portland cement has gradually become one of the most used construction materials for concrete [10,11], being widely used as a construction material due to its availability and easy application process [12,13,14]. It is a vital element in retrofitting structural reinforced concrete (RC) elements because it binds and strengthens the materials, enhancing their durability and load-bearing capacity [15,16,17,18]. Cement substitutes (slag, ground granulated blast furnace slag, fly ash, limestone powder, calcinated clay, ceramic tiles waste, etc.) have been utilized frequently in recent years to improve the properties of concrete and reduce the environmental impact [19,20].

**Figure 1:**
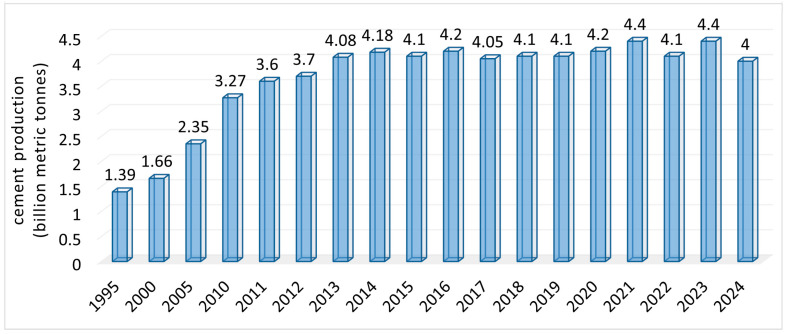
Worldwide cement manufacturing data from 1995 to 2024 [22,23].

The cement demand exploded in 1960 due to the necessity of durable construction materials. The concrete industry uses around 50% of the cement produced, the other half being utilized for plasters, mortars, and blocks [21]. During 1995–2024, the total cement production increased from 1.39 billion metric tons to over 4 billion tons, with peaks of 4.4 billion tons in 2021 and 2023 (Figure 1) [22,23].

**Figure 2:**
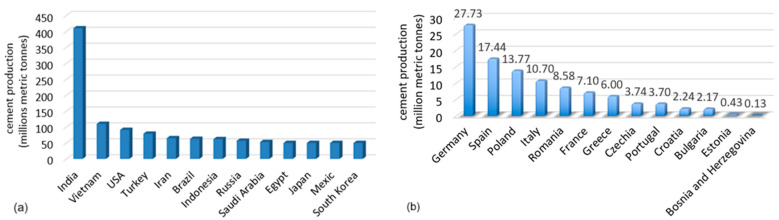
The biggest cement producers in 2024 (**a**) worldwide (without China); (**b**) the biggest manufacturers in European Union. Processed used data available in [22,23,28].

China is the world’s largest cement producer [24], with 1.9 billion metric tons in 2024 (2.1 billion metric tons in 2023 and 2.4 billion metric tons in 2021), i.e., more than half of the world’s production [12,25,26]. The increase in China’s cement production from 1990 was 74% of the worldwide growth [27]. India is the second largest producer in the world, followed by Vietnam, the USA, and Turkey (Figure 2a). In the European Union, Germany is the first-place holder, followed by Spain and Poland (Figure 2b).

Cement production involves processing raw limestone and other raw materials (e.g., shale, clay, etc.) at high temperatures in a kiln to obtain clinker, which is further mixed with gypsum and ground [29]. Clinker fabrication involves [30] the following:

- Collecting the raw material—calcium carbonate (mainly), shale, sand or clay, and bauxite, in small quantities (depending on the receipt).
- Crushing the raw material into pieces about 10 cm in diameter.
- Obtaining the ‘raw meal’ by grinding the raw material.
- Preheating the ‘raw meal’ using the hot gases evacuated from the kiln, in up to six cyclone stages.
- Precalcining, to decompose the limestone.
- Production of the clinker. After precalcining, the ‘raw meal’ goes to the kiln, where the temperature is about 1000 °C. For heating the ‘raw meal’ to about 1450 °C, necessary for clinker formation, a temperature up to 2000 °C should be reached in the rotating kiln, which is obtained by firing the fuel inside it.
- Cooling and storage: The near-molten mixture is rapidly cooled to 100–200 °C.

**Figure 3:**
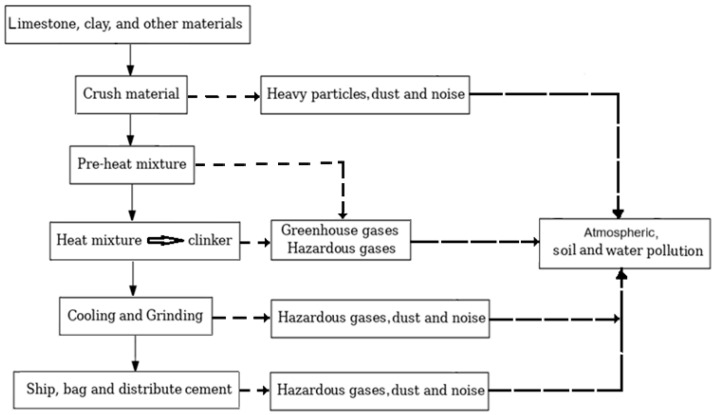
Environmental impact of cement production (adapted from [31]).

Given the complexity of the cement manufacturing process—from raw material extraction and transportation to the final product—numerous sources of pollution emerge, each contributing to potentially harmful environmental impacts. An overview of emissions generated at various stages of cement production is presented in Figure 3.

This review explores the main pollution pathways linked to the cement industry and their effects on the environment and public health. This paper is structured as follows: Section 2 addresses atmospheric pollution and its environmental and health impacts. Section 3 presents key findings on noise pollution and its effects on both the population and the environment. Section 4 and Section 5 examine soil and water pollution, respectively, along with their harmful potential. Section 6 provides concluding remarks, summarizes the main findings, discusses the limitations of the review, and suggests directions for future research.

## 2. Atmospheric Pollution and the Environmental and Health Impacts

### 2.1. Sources of Atmospheric Pollution and the Environmental Impacts

Cement manufacturing is a significant contributor to environmental pollution and anthropogenic climate change, emitting a broad spectrum of atmospheric contaminants, including particulate matter (PM), chlorine gas, nitrogen oxides (NO_x_), sulfur dioxide (SO_2_), ammonia (NH_3_), and various greenhouse gases. Among these emissions, carbon dioxide (CO_2_) is the predominant greenhouse gas driving global warming [32]. Recent studies identified cement production as one of the main industries responsible for CO_2_ emissions [33,34,35,36,37,38,39,40], following the energy sector; every ton of cement produced emits about 0.8–0.9 tons of CO_2_ [35,39]. Furthermore, the industry is estimated to be responsible for approximately 6–8% of total global CO_2_ emissions [33,40]. For example, only in China, CO_2_ production increased from 138 to 818 million metric tons during the period 1993–2019 [27].

**Figure 4:**
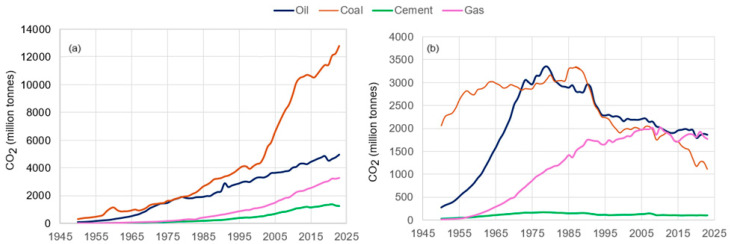
CO_2_ resulting from the combustion of oil, coal, natural gas, and cement industry in (**a**) Asia and (**b**) Europe—processed using data available in [33,40].

The production of lime, the main cement component, involves heating limestone in a rotary kiln, which requires the combustion of fossil fuels to obtain the high temperatures necessary for thermally reducing the limestone [41,42]. Therefore, a significant quantity of the CO_2_ released into the atmosphere originates from this process [43,44]. In the EU, about 60% of emissions result from limestone calcination, about 30% result from reaching high temperatures in the kiln, and about half of the rest result from transportation and electricity consumption [45,46]. Figure 4 compares the emissions resulting from the combustion of coal, oil, and natural gas, alongside emissions from the cement industry in Asia and Europe [33,40], excluding the carbonation sink.

**Figure 5:**
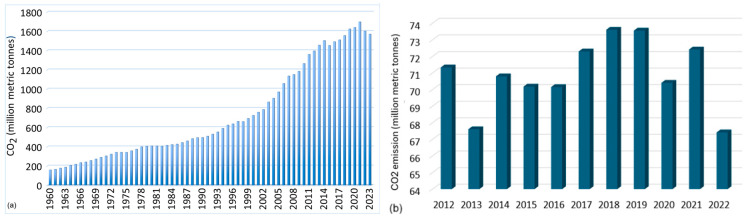
CO_2_ emissions from the cement industry (**a**) worldwide during 1960–2023 [40,47,50,52]; (**b**) in the European Union from 2013 to 2022 [53].

The CO_2_ emissions from the cement industry increased exponentially, especially after 1978, followed by a slight decrease after 2021, when they reached their peak (Figure 5a) [47,48]. In 2019, cement production emitted 2.4 Gt of CO_2_, which accounted for 26% of the total emissions from all industrial sectors [49]. In 2023, the emissions decreased to 1.560 million metric tons of CO_2_, representing approximately 27% of emissions from all industrial sectors [50,51,52]. Within the European Union, the CO_2_ emissions from cement production represent about 4% of the total CO_2_ released. From 1960 to 2023, CO_2_ volume exhibited significant fluctuations, the lowest level being reached in 2022 (Figure 5b) [53].

**Figure 6:**
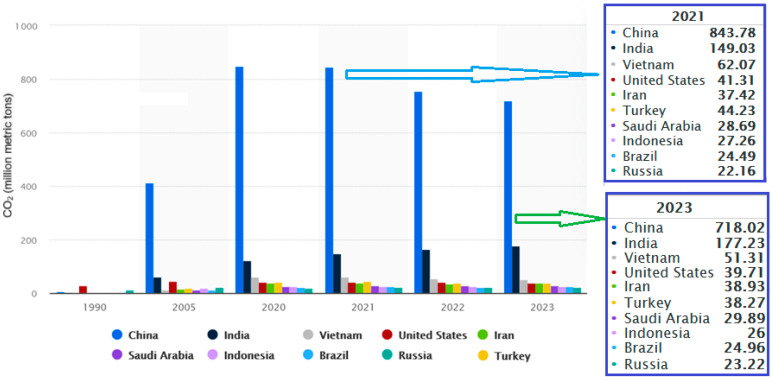
The highest CO_2_ producers from the cement industry (processed using data from [55,56]).

From the viewpoint of the total CO_2_ emissions from cement production per country, after 2005, the first position was constantly occupied by China (718 million metric tons in 2023), followed by India (about 4 times lower than China), Vietnam, and the USA, whose emissions were 4, 14, and 18 times lower than those of China, respectively. There was a variation in the ranking of the top ten countries, determined by the age of the manufacturing facilities and the technologies implemented [54,55]. Figure 6 provides a comparison between the CO_2_ volume emitted in 2021, 2022, and 2023. About 87% of emissions from this industry were issued in China (more than 52%) and developing countries (35%), especially from Asia, in over 2800 production facilities [54].

The cement industry has historically relied on coal and lignite as primary fuel sources due to their high calorific values and widespread availability. These fuels, however, often contain significant levels of sulfur, which, when combusted, can lead to the formation of sulfur oxides (SO_2_) and the buildup of sulfur-rich deposits on kiln rings, causing operational inefficiencies and contributing to air pollution [57,58,59].

Cement kilns, which operate under highly alkaline conditions and reach flame temperatures exceeding 2000 °C, are capable of utilizing alternative high-calorific-value waste fuels. These include used tires, solvents, waste oils, plastics, and hazardous organic wastes such as polychlorinated biphenyls (PCBs) [60,61]. While this co-processing of waste can reduce fossil fuel consumption and offer waste management solutions, it carries significant environmental and public health concerns. When not properly managed, the combustion of these materials can release a range of toxic substances, including particulate matter, (PM), volatile organic compounds (VOCs), polychlorinated dibenzo-p-dioxins (PCDDs), polychlorinated dibenzofurans (PCDFs), hydrogen chloride (HCl), hydrogen fluoride (HF), and heavy metals (e.g., lead, mercury, arsenic, and cadmium) [62,63,64,65,66]. These pollutants can be emitted into the surrounding environment through stack emissions or fugitive releases and pose substantial risks to both ecosystems and human populations.

**Figure 7:**
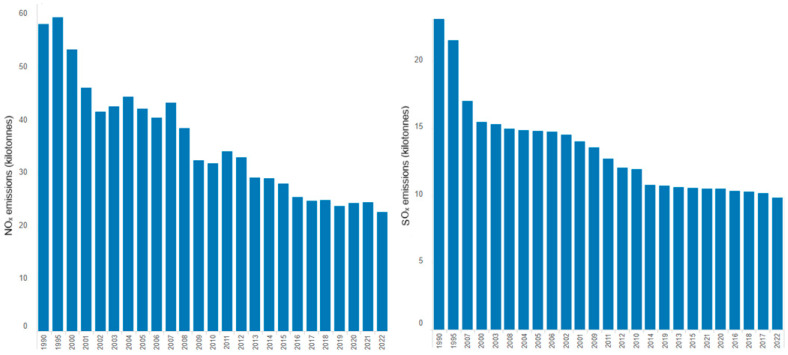
Variation in NO_x_ and SO_x_ emissions from the cement industry in the European Union (processed from [60,68]).

The concentrations of some gases resulting from cement production typically range as follows: SO_2_ < 10–3500 mg/Nm^3^, NO_x_ < 200–3000 mg/Nm^3^, with NO_2_ 5–10% of NO_x_ emissions [67]. Variation in the levels of NO_x_ and SO_x_ emitted in the European Union from cement production during 1990–2022 is represented in Figure 7 [68]. NO_x_ decreased by more than 60% from 1995 to 2022, while SO_x_ emissions declined by about 50%. However, the reduction in NO_x_ and SO_x_ emissions from cement production in European countries, the USA, and Russia did not significantly impact the total emissions due to their increase in Asian countries, particularly China and India [69]. The decline in SO_x_ and NO_x_ emissions from the cement industry reflects the combined effects of regulations (e.g., EU’s Industrial Emissions Directive, US EPA Clean Air Act standards, India’s National Clean Air Programme), the use of cleaner fuels, better technologies (e.g., Improved Combustion and Process Control), and operational efficiencies (e.g., energy efficiency and decarbonization measures), all part of a broader drive toward sustainability and decarbonization.

**Figure 8:**
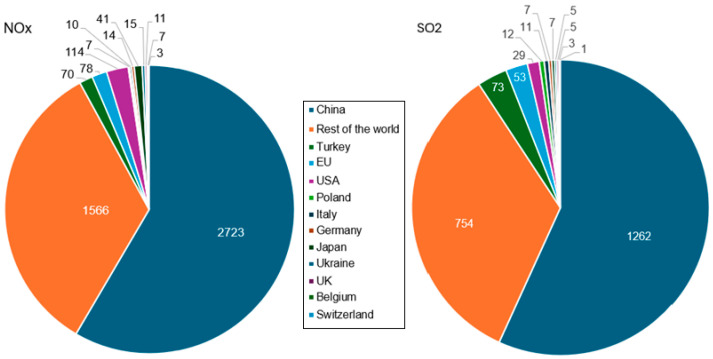
The volume of NO_x_ and SO_2_ emissions (gigatons) from the cement industry in 2019. Processed using the data from [66].

Figure 8 presents a comparison of NO_x_ and SO_2_ emissions (in gigatons) in 2019, in various countries, where the ‘rest of the world’ does not include the listed countries.

China accounts for over 55% of global NO_x_ and SO_2_ emissions, making it the largest producer of both gases. In comparison, the EU emits about 29 times less NO_x_ and 17 times less SO_2_. In 2019, the United States emitted 29 gigatons (114 gigatons) of NO_x_ (SO_2_). Worldwide, the highest NO_x_ emitters are the China National Building Material Group (CNBM), Holcim Group, Anhui Coch (China), Heidelberg Materials, and Cemex (Mexico).

In cement manufacturing, NO_x_ emissions can be categorized into two types, thermal and fuel NO_x_. The first type results from the oxidation of the nitrogen in air, and the second from the nitrogen compounds from the fuel [70]. Thermal NO_x_ mainly results from the combustion zone of the kiln. In other zones, the NO_x_ thermal formation is negligible because the temperatures are less than 1200 °C. NO_x_ emissions vary in the interval [500, 1500] ppm, the highest proportion (95%) being NO, the smallest (less than 1%) being N_2_O, and the rest being NO_2_ [71,72]. NO reacts with oxygen, resulting in NO_2_. Then, by hydrolyzing, it yields HNO_3_ [73].

Neuffer and Laney [70] indicated that during kiln combustion, increasing the air excess (which is typically 5–10%) will increase NO_x_ emissions. Additionally, a slight temperature augmentation above 1430 °C can lead to significant augmentations of the NO level. Moreover, it was shown [74,75] that NO significantly contributes to eutrophication.

The United Nations Global Nitrous Oxide Assessment [76] emphasized that, among greenhouse gases, N_2_O is very stable and is the main gas responsible for destroying the ozone layer and accelerating global warming.

In conclusion, NO_x_ plays a significant role in exacerbating environmental issues such as the formation of ozone in the troposphere, acidification, and eutrophication [77].

SO_3_ and SO_2_ emissions are related to burning fuels that contain sulfur or from the decomposition of the calcium sulfate at high temperatures during clinker manufacturing [78]. According to [79,80], the following reactions are produced:

▪ In the raw mills and preheating zone:

Sulfides + O_2_ → Oxides + SO_2_; Organic S + O_2_ → SO_2_,

▪ In the calcining zone:

Fuel S + O_2_ → SO_2_; CaSO_4_ + C → CaO + SO_2_ + CO,

▪ In the burning zone:

Fuel S + O_2_ → SO_2_; Sulfates → Oxides + SO_2_ + ½ O_2_

As SO_3_ exists as anhydrid, it is easily transformed into SO_2_ and O_2_. The reaction of SO_3_ with alkali and other phases in the kiln tube in the presence of gypsum from raw materials may produce new compounds, such as Ca(Al_6_O_12_)(SO_3_) and Ca_5_(SiO_2_)_2_(SO_4_), K_2_SO_4_, and Na_2_SO_4_ [80,81,82]. A total of 90% of SO_2_ is absorbed in the same zones, based on the following chemical reactions [79]:

▪ In the raw mills and preheating zone:

CaCO_3_ + SO_2_ → CaSO_3_ + CO_2_

▪ In the calcining zone:

CaSO_3_ + ½ O_2_ → CaSO_4_

▪ In the burning zone:

NaO + SO_2_ + ½ O_2_ → NaSO_4_, K_2_O + SO_2_ + ½ O_2_ → K_2_SO_4_

CaO + SO_2_ + ½ O_2_ → CaSO_4_

When water vapor is present in the atmosphere, SO_3_ reacts to form H_2_SO_4_, a major component of acid rain [73,83,84]. This acidic precipitation negatively impacts freshwater ecosystems, harms aquatic life, and impairs plant health and growth by damaging their tissues.

**Figure 9:**
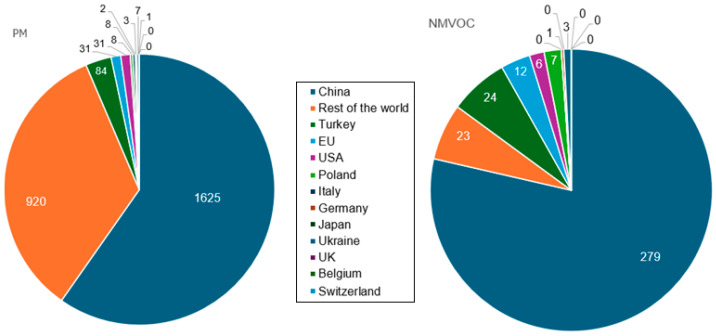
(**left**) M emissions (gigatons) and (**right**) NMVOC from the cement industry in 2019. Processed from the data available in [66].

Particulate matter (PM) is among the most common emissions from the cement industry, followed in a lower proportion by Non-Methane Volatile Organic Compounds (NMVOC). Figure 9 presents the extent of these emissions in 2019. The values of ‘0’ in the pie signify recorded values under 0.5 gigatons.

PM emitted from the cement industry originates from several stages, including quarrying, crushing, grinding, operations in the kiln, packing, landfilled cement kiln dust, etc. [85,86,87]. Although PM generation cannot be avoided, a significant portion can be captured and reused in the production process or recovered and recycled [88]. According to Kalafatoglu et al. [89], studies on dust emissions from various cement plants in Turkey revealed that 44–86% of total emissions were released through the main stacks.

It was also found [90] that the predominant diameters of the PM from the cement industry vary between 0.05 and 5.0 μm. However, the PM’s diameters may vary depending on the technology employed for dust control. For instance, the experiments presented in [86] and cited in [85] have shown that without dust control systems, PM_2.5_ constituted about 7% in wet process kilns and 18% in dry process kilns, while PM_10_ made up 24% and 42%, respectively. When implementing dust control technologies, the proportion of PM_10_ was greater than 80%, and when bag houses were employed, PM_2.5_ represented around 45% of PM emissions. Li et al. [91] also highlighted that the cement industry was responsible for a significant volume of PM_10_ emitted in Shanghai.

Kholodov et al. [92] reported that the PM_10_ emitted from the Spassky factory significantly impacted the air quality in Primorsky Krai and surrounding areas, accounting for 80% of the total particulate matter in the local atmosphere. A study by Smadi et al. [93] in Jordan found that the PM_2.5_ and PM_10_ concentrations were higher than the legal limits in a zone situated less than 300 m from a cement plant. Citing some reports of the Ministry of Ecology and Environment from China, Wang et al. [94] indicated that in 2022, the PM (NO_X_ and SO_2_, respectively) emissions from the cement industry represented 20.9% (17.3% and 5.4%, respectively) of the PM emissions from all industries.

Among the PM emitted during the burning of the fuels to reach the temperatures necessary in the kiln, black carbon can also be released into the atmosphere. This is classified as a short-lived climate pollutant due to its relatively brief atmospheric lifetime, typically spanning only a few weeks before deposition. Despite its transient presence, it exerts a significant influence on the climate system by absorbing solar radiation, thereby contributing to atmospheric warming. Additionally, when deposited on snow and ice surfaces, black carbon reduces their albedo, enhancing solar energy absorption and accelerating cryospheric melt processes [95,96]. Only a few studies considered evaluating its effect, among which was that of Montelongo-Reyes et al. [97], in a study related to the greenhouse and black carbon emissions in Mexico City.

Ammonia (NH_3_) emissions from the cement industry have become an increasing concern, particularly due to the large-scale use of selective non-catalytic reduction (SNCR) systems for NO_x_ control. Moreover, ammonia may also be present in raw materials and fuels. While NH_3_ is partially removed during the cement production process—mainly through absorption onto the alkaline components of the raw meal and oxidation to NO_x_ in the calciner and kiln—these mechanisms may become insufficient when ammonia concentrations are high. Emissions are increased when the raw meal grinding system is inactive, as exhaust gases bypass the mill, which is the primary zone where NH_3_ is absorbed [98].

A study of 14 cement lines in China reported an average NH_3_ concentration of 35.29 mg/Nm^3^, with more than 84% of measurements above the admissible limit [99]. Emission levels were closely linked to the amount of ammonia injected and the operational status of raw mills. The resulting average NH_3_ emission factor surpassed the U.S.’s recommended guideline of 0.066 kg/t [100].

NH_3_ utilized for NO_x_ reduction can result in residual amounts being retained in combustion byproducts, particularly fly ash [101,102,103]. Studies by Spanka and Thielen [102] found that the concentrations of NH_3_ in fly ash varied from 8 to 130 mg/kg. When the proportion of fly ash in concrete was 20%, up to 30% of the ammonia content may be released in less than one week [104].

Similarly, gypsum produced from limestone slurry during flue gas desulfurization may also contain ammonia, posing another potential source. These findings indicate that both amines and ammonium compounds can be released from materials derived from emission control processes [102,104]. Another potential emission source of ammonia is gypsum generated from limestone slurry in flue gas desulfurization [104].

### 2.2. Health Impact of Atmospheric Pollution from Cement Manufacturing

A considerable number of studies indicate that at a global scale, the CO_2_, NO_x_, SO_2_, and PM emitted in the atmosphere have a greenhouse effect, leading to stratospheric ozone depletion, acid rain formation, biodiversity loss, and reduced agricultural crop productivity. Considering the direct impact on the population, air pollution has been linked to various respiratory problems (e.g., asthma, bronchitis, and tuberculosis) and other health issues, such as eye irritation, heart disease, and premature deaths [105,106,107,108,109,110,111,112,113,114]. Here, we emphasize some of them.

CO_2_ is not a direct pollutant; concentrations up to 1000 ppm are considered safe for human health in indoor environments. However, experimental studies have demonstrated that sustained exposure to elevated levels of CO_2_ can have detrimental effects on human health [115,116,117,118,119,120,121,122,123]. Chronic CO_2_ exposure has been associated with reduced cognitive performance, oxidative stress, bone demineralization, kidney calcification, etc. [118,121]. Beyond human health, elevated CO_2_ concentrations also exert harmful effects on ecosystems, adversely impacting plant and animal life, as documented in [124,125].

NO_x_ released into the atmosphere poses both direct and indirect risks to human health. Nitrogen dioxide, in particular, is a respiratory irritant capable of penetrating deep into the lungs. Short-term exposure—approximately 15 ppm—can cause throat irritation and coughing, exacerbate asthma symptoms, and disrupt pulmonary function, especially at concentrations exceeding 25 ppm [72,84]. Prolonged exposure has been associated with the development of chronic bronchitis, increased susceptibility to respiratory infections among vulnerable populations such as the elderly and children, and adverse effects on normal childhood development [126]. Indirectly, NO_x_ exposure may exacerbate pre-existing cardiovascular conditions [106,114,127,128,129].

In 2020, about 10.6% of the population was affected by chronic obstructive pulmonary disease (COPD). It represents about 480 million persons all over the world [130]. Many studies indicate an augmentation of COPD at high concentrations of NO_2_ and O_3_ [110] and significant dependence on its occurrence on the PM_2.5_ presence [131,132]. Lang et al. [129] detected correlations between cancer and heart disease cases and exposure to high NO_x_ concentrations for long periods.

As reported in [105], fine particulate matter (PM_2.5_) and ozone (O_3_) are considered among the most hazardous air pollutants to human health due to their ability to penetrate deep into the respiratory system and enter the bloodstream. Harrison and Yin [133] emphasized that, in addition to chemical composition, particle size is a critical determinant of particulate matter toxicity. Smaller particles exhibit a greater likelihood of reaching the alveolar regions of the lungs and subsequently entering the circulatory system [134]. The health effects of PM exposure range from respiratory and ocular irritation, infections, and airway inflammation to more severe conditions such as sinusitis, bronchitis, asthma, cardiovascular diseases (including atherosclerosis, hypertension, myocardial infarction, and heart failure), and various forms of cancer. PM has also been linked to cellular-level damage, including oxidative stress, mitochondrial dysfunction, and lipid peroxidation [108,112,129,131,132,133,135,136,137]. Furthermore, exposure to elevated levels of PM has been associated with reduced infant birth rate and increased mortality [138,139,140].

**Figure 10:**
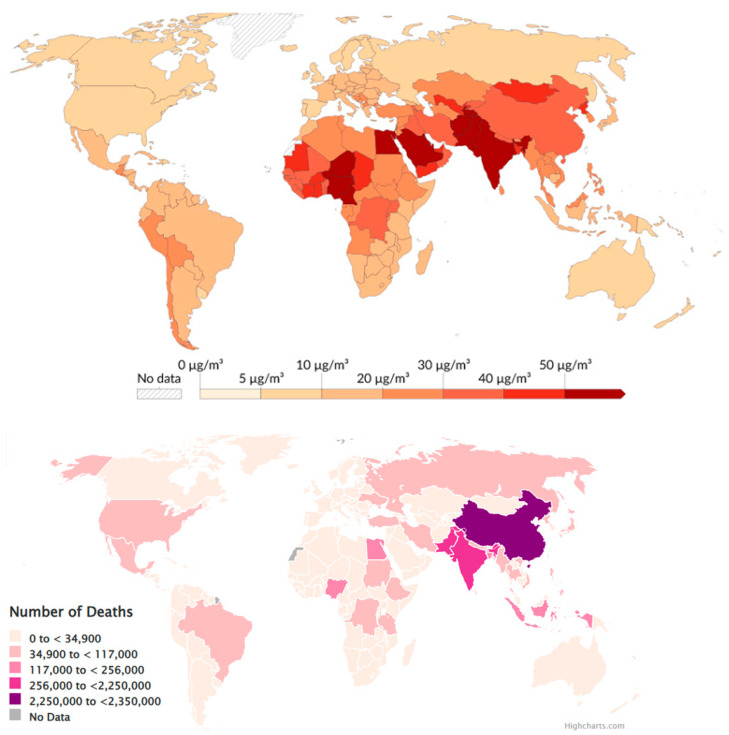
(**top**) Exposure to PM in 2019 (from https://ourworldindata.org/grapher/pm25-air-pollution?time=2019) (accessed on 1 June 2025).; (**bottom**) number of deaths in 2021 due to atmospheric pollution (from https://www.stateofglobalair.org/hap) (accessed on 1 June 2025).

In a study of Europe, Juginović et al. [141] reported that among 368,006 life losses caused by pollution in 2019, more than 90% were due to long-term exposure to PM_2.5_. Figure 10 (top) presents people’s estimated exposure to PM-related in 2021, while Figure 10 (bottom) contains a regional map illustrating the life losses due to outdoor PM air pollution for the same year.

The highest PM pollution from the cement industry was registered in Asia (especially India), Gulf countries, and Western Africa, but the highest mortality associated with PM pollution was found in China and India.

A study conducted in Doroud, Iran [142] found the highest concentrations of PM_10_ at distances of 1600–1800 m from a local cement factory, downwind. Their values significantly exceeded the World Health Organization (WHO) air quality guidelines, posing serious health risks to factory workers and populations from neighboring settlements. Based on the findings, a distance of at least 7.5 km from the factory was recommended to ensure a safe level of exposure for public health.

Wahas et al. [143] detected high concentrations of PM_2.5_ and PM_10_ in some cement factories in Haripur (Pakistan). The biggest mean concentrations of PM_10_ and PM_2.5_ were 1552 and 7867.5 µg/m^3^, identified in the factory’s main crusher and, respectively, at the cement mill. De Souza Zorzenão al. [144] found that the limits imposed by WHO for PM_2.5_ were exceeded near a cement factory in Brazil between 2021 and 2022, indicating 3.5% (4.3% and 4.7%, respectively) estimated years of life lost. After analyzing the samples collected in the vicinity of two cement facilities in various seasons, Sánchez-Soberón et al. [145] found the highest concentrations of PM_1_, PM_2.5_, and PM_10_ in the cold season, with the biggest risk (non-carcinogenic and carcinogenic) for PM_1_.

In a study related to a cement plant in Kashmir (in 2011), Mehraj et al. [146] reported that most people living in the facility neighborhood presented allergies and eye irritation, breathing difficulties, asthma, chronic bronchitis, irregular heartbeat, chest pain, etc. Lung cancer affecting 1% of the inhabitants of that area was also documented.

A study conducted in Spain over 10 years [147] reported the highest cancer-related mortality percentage among the population living in zones around cement factories, with the colon–rectum and pleura being the most prominent cancer types. Koh et al. [148] explored the possible correlation between cement dust exposure and cancer risk among male workers in six Korean Portland cement factories. They identify a significantly higher incidence of stomach cancer among workers from the production sector. Although lung cancer mortality was also higher, the association was not statistically significant. The findings suggest a possible link between cement exposure—particularly to hexavalent chromium—and increased cancer risk.

Further evidence comes from a cross-sectional study by Lee et al. [149], which found a higher prevalence of emphysema among individuals more exposed to cement plant emissions, suggesting adverse respiratory effects on nearby residents. Similarly, research on ventilation impairment in a Korean community near a cement facility [150] reported compromised lung function. In a study from the same country, Eom et al. [151] found that the incidence of bronchus and lung cancer was notably higher in the study sample, especially among men, compared to the incidence officially reported. They observed a marginal increase in laryngeal cancer in men and salivary gland cancer in women residing in the vicinity of the cement factory. Etim et al. [109] provided an extensive review of the research on pollution from the cement industry in Nigeria and the impact on the environment and the population’s health, emphasizing the necessity of taking urgent measures to reduce emissions.

## 3. Noise Pollution and Its Impact on Environment and Population

### 3.1. Noise Pollution and Its Impact on the Environment

Noise pollution is recognized as a significant environmental concern in areas where cement is produced. The noises in cement plants arise from three principal sources. Mechanical noise is issued when machines (mills, crushers, and collectors) function in the process of grinding the raw material. The electromagnetic noise results from the functioning of the electrical motors [152]. The third kind of noise is the result of the gas dynamics [153].

The noise levels in cement plants, particularly during raw material preparation and processing, generally range from 68.8 to 103.3 dBA. Zhu et al. [154] found higher noise levels in the grinding station (89–105 dBA), exceeding the admissible value of 85 dBA per eight working hours. Mndeme and Mkoma [155] found that in a plant in Tanzania, the noise level varied from 50 dBA to 104.82 dBA in the offices and production sectors. The highest noise was recorded in the power plant, followed by the compressor room (96.67–102.02 dBA), raw mill (92.82–96.48 dBA), and limestone crusher (83.73–93.40 dBA). In a study from China, Zhang et al. [156] reported that in the ball mill and crusher rooms, raw material, and coal mill rooms, the noise was above 100 dBA. In all the other sections, it remained between 85 and 100 dBA. Similar values were determined by Ali et al. [157] and Noorpoor and Orkomi [158] in cement factories in Rabak, Sudan, and Tehran, Iran, respectively.

Generally, cement factories are situated outside cities and villages to avoid a negative impact on the population as much as possible. However, Sordello et al. [159] noted that the noise generated by cement plants negatively impacts local biodiversity. The noise from blasting and other machinery leads to changes in wildlife behavior and reduces the availability of habitats for wildlife. Many species, like birds and mammals, avoid areas of high noise. Furthermore, noise pollution directly impacts wildlife’s communication and reproductive behaviors, contributing to a decline in local ecosystems [160,161]. Pollinators have been shown to decrease their activity in noisy areas, which could hinder agricultural productivity [162].

### 3.2. Health Impact of Noise Pollution from Cement Manufacturing

Noise pollution originating from cement plants poses serious risks to human health, not only to the environment [156]. Prolonged exposure to high noise levels, particularly those exceeding 80 dB(A), as in the cement factories, can lead to significant health issues, including noise-induced hearing loss (NIHL) [153,156,163].

Thai et al. [153] indicated that NIHL was prevalent (in 52% of cases in their study) when sound levels reached around 100–105 dB(A). Hernández-Gaytán et al. [164] reported that the highest noise was recorded in the packer job post, and the largest number of workers with various levels of hearing issues worked in the calcination sector. Moreover, more than half of the study sample suffered from partial hearing loss. Ali et al. [165] showed that more than 12% of people employed in a cement factory experienced moderate hearing loss in the right ear, and two-thirds suffered mild hearing loss in the left ear.

Workers in cement factories for long periods often experience a variety of symptoms, such as irritability, whistling/buzzing, headaches, fatigue, dizziness, insomnia, increased blood pressure, and memory loss [155,166,167,168]. Additionally, continuous exposure to excessive noise may result in neurasthenia, which affects mental and physical well-being [153,156,169]. Even in a mildly noisy environment, workers do not hear clearly and are not able to recognize warning signals that could pose a safety risk [153]. Therefore, they are more exposed to work accidents [169,170,171].

## 4. Soil Pollution from the Cement Industry and Its Impact on Environment and Population Health

### 4.1. Soil Pollution from Cement Industry

Numerous studies have confirmed the presence of heavy metal contamination in soils near cement factories [172,173,174,175,176]. For instance, lead (Pb) and zinc (Zn) were found at moderately elevated levels in surface soils near cement plants in Nigeria [172]. In Saudi Arabia, concentrations of Pb, Cr, Zn, Ni, and Cu exceeded regulatory limits near a cement factory [173]. Cadmium (Cd), chromium (Cr), and nickel (Ni) were also detected at concerning levels in Nigerian soil samples near cement facilities [174]. Egbe et al. [175] reported increased levels of Cr, Cu, Fe, Mn, Pb, and Zn close to a cement plant, indicating moderate contamination. Maina et al. [176] observed heavy metal enrichment in soil—2 to 10 times higher than in uncontaminated areas—reaching a maximum at 5–7 km from the Ashaka cement plant in Nigeria. In Pakistan’s Punjab region, Ismail et al. [177] detected contamination with various heavy metals within 500 m of three cement plants, based on the geoaccumulation index and concentration factor. In Spain, Schuhmacher et al. [178] and Rovira et al. [179] found increased levels of heavy metals in both soil and vegetation, especially during the cold season. Olatunde et al. [180] assessed health risks due to heavy metal accumulation in soils around the Ibese cement plant.

Cement production also leads to elevated particulate matter (PM) deposition. Soussia et al. [181] recorded extremely high levels of dust deposition—above regulated limits—especially in the outdoor areas of a cement factory, with peaks in December and January. In Nigeria, increased PM deposition and soil alkalinity were also observed in the vicinity of cement production sites [182].

Several studies report changes in soil physical and chemical properties near cement factories. For example, Anurag et al. [183] found modified soil properties 500 m downwind of a cement plant in Chhattisgarh, India, including elevated organic carbon, silt, and sand content. Lamare and Singh [184] documented increases in soil alkalinity, organic carbon, electrical conductivity, and water retention capacity near the Meghalaya cement plant in India. Uwasu et al. [38] highlighted that waste and slurry disposal during any cement manufacturing process (dry, semi-dry, and wet) contributes to soil degradation and environmental harm. Jadaoon et al. [185] found that the dust and pollutant deposition reduced chlorophyll content in plants, along with decreases in height and stem diameter, indicating impaired plant growth in the vicinity of cement factories.

### 4.2. Impact of Soil Pollution with Heavy Metals from Cement Industry on Public Health

Soil contamination with heavy metals poses a threat to public health, particularly in regions impacted by industrial activities such as cement manufacturing. Heavy metals are non-biodegradable, tend to accumulate in soils, and can enter the human body through multiple exposure routes: the ingestion of contaminated food or water, inhalation of resuspended dust particles, and dermal contact with polluted soils. Once absorbed, many heavy metals exert toxic effects even at low concentrations, often leading to chronic diseases and systemic dysfunctions.

Lead is among the most toxic environmental pollutants, with well-documented effects on multiple organ systems. Chronic exposure, particularly in children, is associated with neurodevelopmental disorders, reduced intelligence quotient, attention deficits, and behavioral issues. In adults, prolonged exposure may contribute to renal dysfunction, hypertension, reproductive issues, and anemia. Lead interferes with enzymatic functions and calcium-mediated neuronal signaling, making young children and pregnant women especially vulnerable to its neurotoxic effects [186].

Cadmium is primarily absorbed through ingestion or inhalation and accumulates in the kidneys and bones. Long-term exposure is known to cause nephrotoxicity. It also interferes with calcium metabolism, leading to bone demineralization and diseases such as osteoporosis and osteomalacia. Cadmium has been classified as a Group 1 human carcinogen by the IARC due to its role in inducing lung and prostate cancer [187]. The toxicological reference value for this element is 0.21–0.36 µg/(kg body weight per day).

Hexavalent chromium [Cr(VI)] is highly toxic. It penetrates biological membranes and induces oxidative stress, DNA damage, and apoptosis. Inhalation of Cr(VI)-containing dust can cause severe respiratory disorders, including chronic bronchitis, nasal septum perforation, and lung cancer. Ingestion may lead to gastrointestinal irritation and systemic toxicity affecting the liver and kidneys [188,189]. The Tolerable Upper Intake Level for adults is 10 mg/day, but chronic toxicity can occur at 40–50 mg/kg. 

Toxicity from nickel can occur through various routes of exposure, including inhalation, ingestion, injection, and dermal absorption. Acute inhalation may cause respiratory inflammation, including pneumonitis and, in extreme cases, acute respiratory distress syndrome. Case reports have shown nickel nanoparticles to deposit in alveolar macrophages, triggering lung injury and systemic inflammation [190,191]. Chronic exposure may lead to allergic contact dermatitis and has been implicated in nasal and lung cancers due to its genotoxic potential [192,193]. The ingestion of nickel salts will acutely manifest toxicity. Gastrointestinal symptoms are prominent, including nausea, vomiting, diarrhea, abdominal pain, and hepatic dysfunction [194]. Dermal absorption can lead to type IV skin sensitivity, causing pruritus and erythematous papules [195].

Workplace standards for nickel compounds (measured as nickel) vary. According to OSHA, the limit of Cu exposure is 1 mg/m^3^ (8 h TWA). NIOSH established 0.015 mg/m^3^ (10 h TWA) and ACGIH, 0.2 mg/m^3^ (8 h TWA) [196]. 

Although zinc and copper are essential micronutrients, excessive environmental concentrations due to industrial contamination may result in toxicity. Elevated zinc intake can disrupt copper metabolism, causing gastrointestinal distress, immune dysfunction, and anemia. Excessive copper exposure may lead to hepatic and neurological damage, especially in individuals with Wilson’s disease, a genetic disorder affecting copper excretion [197,198].

Populations residing near cement factories or other industrial facilities face elevated exposure risks. Children are particularly vulnerable due to their behaviors (e.g., playing outdoors and hand-to-mouth activity) and greater absorption rates. Contaminated soils can also lead to indirect exposure through the food chain, especially in agricultural zones where crops absorb heavy metals from polluted soil.

## 5. Water Pollution from the Cement Industry and Its Impact on Environment and Population Health

According to van Oss and Padovanni [86], the principal concern about water use in the cement industry is its quality [199] and adequate supply. Water is employed at multiple stages of the cement production process. Directly, water is utilized in washing the raw materials before processing and as a facilitator of raw materials grinding. Various particles (e.g., lime, aluminum oxides, and iron) are washed, leading to water contamination [154]. Indirect use involves cooling kiln bearings, grinding equipment, thermal piping, compressors, etc.

Several studies have investigated the effects of cement wastewater on the water quality of rivers that receive such effluents [200,201,202,203] and provided evidence that water bodies may be contaminated by hazardous substances originating from cement manufacturing facilities. An analysis by Mbaka [204] found that the indicators of water quality of the Athi River (pH, electrical conductivity, turbidity, total suspended and dissolved solids, temperature, and pH) were above the imposed limits, as was the Pb concentration. These results are consistent with [205], indicating that possible surface water contamination may arise from liquid waste and effluent from quarry operations, the press house, and milling processes during cement production.

Additional contamination pathways occur through the leaching of the cement kiln dust (CKD) landfill. Every metric ton of clinker generates approximately 50–150 kg of CKD waste [206], but part is reused in cement manufacturing. Soluble contaminants released during precipitation can increase the heavy metal concentrations in nearby groundwater. Environmental consequences arise from both immediate and long-term effects.

Limited research has been conducted on how aquatic organisms respond to the discharge of cement effluents. A study conducted by Olaleye and Oluyemi [207] in tropical regions found a decline in plankton species richness and diversity in areas surrounding cement factory catchments. Arimoro et al. [208] emphasized the extinction of some macroinvertebrate taxa in the river where the wastewater from a cement plant was permanently evacuated. Oyinlola et al. [209] identified elevated levels of Cd, Cr, and Pb in local rivers downstream of the Ewekoro Cement Factory in Nigeria. They also detected significant abnormalities in the African catfish grown in the river where the wastewater from the Ewekoro Cement Factory was discharged.

Long-term studies of rivers adjacent to cement plants show an average of 40–60% lower macroinvertebrate diversity, with a negligible amount of EPT (Ephemeroptera, Plecoptera, Trichoptera) taxa (<1 individual collected) collected within 2 km from discharge [210]. Alkaline pH shocks after discharges may result in 80–100% mortality of organisms sensitive to the change in pH (e.g., Daphnia magna) within 96 h [211].

This water contamination was associated with alterations in fish histopathology, affecting the gills, kidneys, and liver. The consumption of such fish and drinking improperly treated water from these rivers can produce chronic health effects in humans, like kidney and liver damage or digestive issues [109,209]. However, more studies must clarify the impact of the consumption of contaminated water from the cement industry on the population’s health.

## 6. Conclusions

The cement industry, while vital to infrastructure and economic development, poses significant environmental challenges through its multifaceted pollution outputs. This review has highlighted how atmospheric emissions—particularly CO_2_, NO_x_, SO_2_, and PM—contribute to air quality degradation and climate change. Simultaneously, cement production affects soil health through the deposition of heavy metals and alkaline dust, leading to reduced fertility and ecological imbalances. Water resources are not spared, with cement effluents and slurry runoff introducing suspended solids and toxic elements into nearby water bodies, impairing aquatic ecosystems. Additionally, sonic pollution from quarrying, grinding, and transportation operations generates persistent noise that disrupts both human and wildlife populations.

Researchers have extensively documented the environmental impact of cement production, emphasizing its contribution to atmospheric, soil, water, and sonic pollution. Numerous studies have also proposed a variety of solutions aimed at mitigating these issues. Given the breadth of existing research and the extensive body of literature addressing different aspects of the problem, we believe this review serves as a foundation for further work focused specifically on mitigation strategies and reducing the environmental impact. Accordingly, a follow-up review will be dedicated to discussing the range of solutions that have been proposed or implemented to reduce pollution from the cement industry. In addition, the effectiveness of these measures will be critically assessed in a subsequent article.

Given the scale and complexity of the environmental challenges posed by the cement industry, there is an urgent need for more stringent regulations, the adoption of cleaner technologies, and the implementation of sustainable practices throughout the production lifecycle. Innovations such as carbon capture, waste heat recovery, the use of alternative fuels, and precision environmental monitoring offer promising pathways to reduce the industry’s footprint. Ultimately, addressing this global issue will require coordinated efforts and collaboration among industry stakeholders, policymakers, and the scientific community to ensure a balance between development and ecological sustainability.

## Data Availability

No new data was created.
